# Rethinking the Role of the Renin-Angiotensin System in the Pandemic Era of SARS-CoV-2

**DOI:** 10.3390/jcdd10010014

**Published:** 2023-01-01

**Authors:** Fabio Angeli, Martina Zappa, Paolo Verdecchia

**Affiliations:** 1 Department of Medicine and Surgery, University of Insubria, 21100 Varese, Italy; 2 Department of Medicine and Cardiopulmonary Rehabilitation, Istituti Clinici Scientifici Maugeri IRCCS, 21049 Tradate, Italy; 3 Fondazione Umbra Cuore e Ipertensione-ONLUS, and Division of Cardiology, Hospital S. Maria della Misericordia, 06100 Perugia, Italy

After assessing the levels of spread and severity of the severe acute respiratory syndrome coronavirus 2 (SARS-CoV-2) infection, academic literature focused on the pathophysiology of coronavirus disease 2019 (COVID-19) [1,2,3]. The *Journal of Cardiovascular Development and Disease* has actively contributed to the growth of our knowledge in this field, with articles spanning a range of topics from mechanisms and risk stratification to therapeutic interventions [4,5,6,7].

It is well documented that SARS-CoV-2 infection may trigger a cascade of systemic events affecting various organs and tissues [8,9]. Emerging data support that the leading actor in the pathogenesis of the complications of COVID-19 is a dysregulation of the renin–angiotensin system (RAS) [10,11,12]. Related evidence from experimental and clinical studies have been accrued [12] and the imbalance between angiotensin II (Ang II) and Angiotensin_1–7_ (Ang_1–7_) caused by the interaction between SARS-CoV-2 (as mediated by the binding of the Spike protein of the virus) and the angiotensin converting enzyme 2 (ACE_2_) receptors exerts a pivotal role on the clinical picture and outcome of COVID-19 [10,11,12,13]. More specifically, SARS-CoV-2 entry into human cells is mediated by the efficient binding of the Spike protein to ACE_2_ [14,15]. The ACE_2_ is a trans-membrane type I glycoprotein (expressed in almost all human tissues [16]) which uses a single extracellular catalytic domain to cleave an amino acid from Ang II to form Ang_1–7_ [17]. The viral entry process consists of three main steps [14,18]. In the first step, the N-terminal portion of the viral protein unit S1 binds to a pocket of the ACE_2_ receptor [14]. In the second step, the protein cleavage between the S1 and S2 units is operated by the receptor transmembrane protease serine 2 (TMPRSS2) which facilitates viral entry and downregulates surface ACE_2_ expression [19]. In the third step, the viral S2 unit undergoes a conformational rearrangement after the cleavage of the viral protein by TMPRSS2, driving the fusion between the viral and cellular membrane and promoting the entry of the virus into cell, release of its content, replication, and infection [20].

The failure of the counter-regulatory RAS axis, characterized by the decrease in ACE_2_ expression and generation of the protective Ang_1–7_, is strictly implicated in the development of severe forms of COVID-19 [10,11,13,14,21,22]. ACE_2_ internalization, downregulation, and malfunction, predominantly due to viral occupation, dysregulates the protective RAS axis with increased generation and activity of Ang II and reduced formation of Ang_1–7_ [10,11,13] (Figure 1). This has been corroborated by recent investigations supporting the evidence of the development of an “Ang II storm” [23] or “Ang II intoxication” [24] during the SARS-CoV-2 infection [7,11,12,13,25,26]. Ramos and co-workers provided a new assessment of available data, exploring COVID-19 as a molecular disease that causes negative regulation of ACE2 and RAS with microcirculatory changes responsible for the wide variety of injury mechanisms observed in different organs as a result of the disease [23]. Similarly, Sfera and co-workers proposed a common pathophysiological denominator for COVID-19 [24]. They hypothesized that the outlook of COVID-19 is negatively correlated with the intracellular accumulation of Ang II promoted by the viral blockade of its degrading enzyme receptors [24]. Notably, similar effects of COVID-19 vaccines have been recently postulated [8,27]. Indeed, COVID-19 vaccines increase the endogenous synthesis of SARS-CoV-2 Spike proteins. The free-floating Spike proteins synthetized by cells targeted by vaccines and destroyed by the immune response may massively circulate in the blood and systematically interact with ACE_2_ receptors, thereby promoting ACE_2_ internalization and degradation [8,27]. In other words, these reactions might result in pathological features which resemble those of SARS-CoV-2 infection [8,27].

The reduced catalytic efficiency of ACE_2_ resulting from viral occupation and down-regulation of these receptors may be particularly detrimental in patients with baseline deficiency of ACE_2_ receptor activity [11,12]. Notably, phenotypes of ACE_2_ deficiency include advanced age, some cardiovascular risk factors, and previous cardiovascular events [9,10,11,12,28,29,30,31,32,33,34,35,36,37]. Aging is associated with declining levels of ACE_2_ expression in experimental and human models [38,39,40,41]. Chen and co-workers analyzed GTEx and other public data in 30 tissues across thousands of individuals and they found an age-dependent decrease in ACE_2_ expression in all ethnic groups [40]. Furthermore, human and mouse data analysis revealed that ACE_2_ expression is reduced in type 2 diabetes [40]. Similar results have been also obtained in other reports showing that diabetes mellitus is associated with a reduction in ACE_2_ expression and with Ang_1–7_-generating system downregulation [42,43].

Hypertension is associated with RAS over-activation, increased angiotensin type 1 receptor (AT_1_R) stimulation by Ang II, and downregulation of ACE_2_ [44].

ACE_2_ deficiency is also documented in several experimental models of cardiovascular complications, including congestive heart failure, myocardial ischemia and infarction, and coronary artery disease [45,46,47,48,49]. For example, Kassiri and co-workers reported that loss of ACE_2_ facilitates adverse post-myocardial ventricular remodeling through potentiation of Ang II effects [48]. In their experimental analyses, myocardial infarction was associated with a persistent increase in ACE_2_ protein in the infarct zone in wild-type mice, whereas loss of ACE2 enhanced the susceptibility to myocardial infarction, with increased mortality, infarct expansion, and adverse ventricular remodeling characterized by ventricular dilation and systolic dysfunction [48]. In ACE_2_-deficient hearts, elevated myocardial levels of Ang II and decreased levels of Ang_1–7_ in the infarct-related zone were associated with increased production of reactive oxygen species [48]. Additionally, ACE2 deficiency leads to increased matrix metalloproteinase (MMP) 2 and MMP9 levels with MMP2 activation in the infarct and peri-infarct regions, as well as increased gelatinase activity leading to a disrupted extracellular matrix structure after myocardial infarction. Moreover, loss of ACE_2_ was also associated with increased neutrophilic infiltration in the infarct and peri-infarct regions, resulting in upregulation of inflammatory cytokines [48].

However, ACE_2_ are not the exclusive angiotensinases in humans. Other angiotensinases involved in the processing of Ang II to Ang_1–7_ may influence the detrimental interactions between Spike proteins of SARS-CoV-2 and ACE_2_ receptors [50,51,52,53]. As recently suggested, the relative activity of different angiotensinases (including ACE2, prolyl carboxypeptidases, and prolyl oligopeptidases) and their changes in the cardiovascular continuum disease, should be taken into consideration to fully understand the pathogenesis of COVID-19. In other words, different mechanisms of Ang II cleavage and accumulation may be involved in a unique pathophysiological mechanism explaining the risk of the progression to severe forms of COVID-19 [8,12].

In conclusion, understanding the pathophysiology of COVID-19 influences the treatment of these patients. Importantly, some hypotheses have been made on the potential therapeutic approach of restoring the ACE2/Ang_1–7_ pathway.

Pharmacological modulation of RAS may be useful to enhance the blockade of the transition from infection to severe forms of COVID-19 [25]. In a recent prospective study of hypertensive patients with COVID-19, we documented that exposure to RAS modifiers was associated with a significant reduction in the risk of in-hospital mortality compared to other blood pressure-lowering strategies [7]. Exposure to ACE-inhibitors was not significantly associated with a reduced risk of in-hospital mortality compared with patients who were not treated with RAS modifiers [7]. Conversely, angiotensin receptor blockers users showed a 59% lower risk of death (*p* = 0.016) even after allowance for several prognostic markers, including age, oxygen saturation, occurrence of severe hypotension during hospitalization, and lymphocyte count [7].

Moreover, the delivery of functional soluble ACE_2_ forms may trap the virus and stimulate the RAS protective pathway [13]. Finally, ACE_2_ inhibitors may block or attenuate the binding of SARS-CoV-2 Spike protein to the pocket of the ACE_2_ [13]. This pharmacological approach could reduce viral internalization into ACE2-expressing cells [13]. However, pharmacological inhibition of ACE_2_ may exert enzymatic activities with or without inactivation of ACE_2_. The real challenge in the field of ACE inhibition is to modulate SARS-CoV-2 binding to ACE2 without blocking the protective conversion of Ang II into Ang_1–7_ [13].

## Figures and Tables

**Figure 1:**
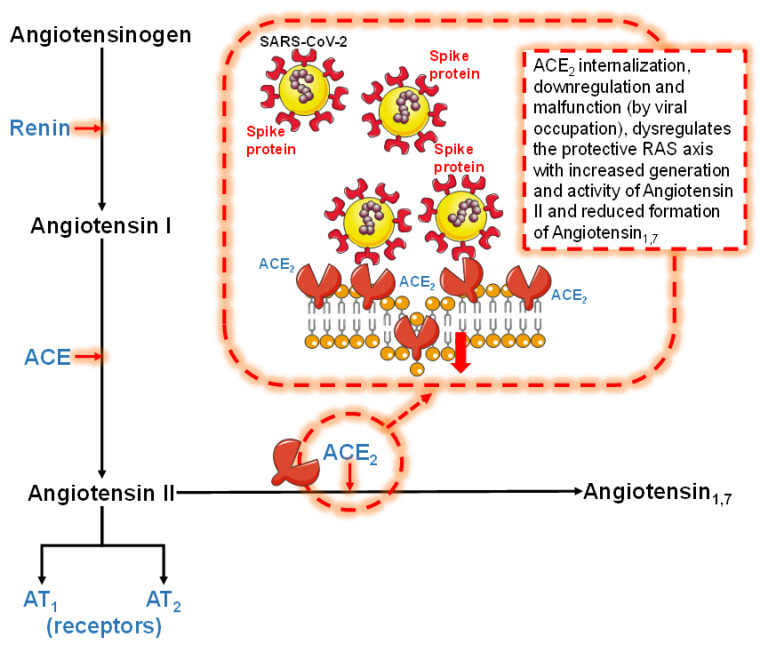
Schematic representation of the renin–angiotensin system (RAS, left side). The effects of viral occupation (right side) on the failure of the counter-regulatory RAS axis are also depicted (see text for details). ACE = angiotensin converting enzyme, ACE_2_ = angiotensin converting enzyme 2, AT_1_ = angiotensin II receptor type 1, AT_2_ = angiotensin II receptor type 2, RAS = renin-angiotensin system, SARS-CoV-2 = severe acute respiratory syndrome coronavirus 2.
